# Probiotics, prebiotics, and synbiotics for the treatment of asthma: Protocol for a systematic review

**DOI:** 10.1097/MD.0000000000017840

**Published:** 2019-11-22

**Authors:** Ling Huang, Jing Guo, Wenyuan Li, Mei Jiang, Fei Wang, Jia Kang, Tiegang Liu, Xiaohong Gu

**Affiliations:** a Beijing University of Chinese Medicine, Beijing; b Chengdu University of Traditional Chinese Medicine, Affiliated Hospital of Chengdu University of Traditional Chinese Medicine, Chengdu, Sichuan; c Gulou Hospital Affiliated to Capital Medical School, Beijing, China.

**Keywords:** asthma, prebiotics, probiotics, protocol, synbiotics, systematic review

## Abstract

**Background:**

Asthma is a common chronic disease with heavy burden. The number of asthma patients may continue to grow in the next 10 years. Existing conventional treatments have problems in which a small number of patients do not respond, often accompanied by side effects, or are too expensive. Probiotics, prebiotics, and synbiotics have been widely used in allergic and inflammatory diseases including asthma. However, their effectiveness and safety are still obscure and deserve further investigation.

**Objective:**

To assess the effect and safety of probiotics, prebiotics, and synbiotics in treating asthma.

**Methods:**

We will summarize and meta-analyze randomized controlled trials (RCTs) of probiotics, prebiotics, and synbiotics for the treatment of asthma. RCTs comparing probiotics, prebiotics, and synbiotics with blank control, placebo, or conventional therapies will be included. RCTs comparing probiotics, prebiotics, and synbiotics plus conventional therapies with conventional therapies alone will also be included. The following electronic databases will be searched: PubMed, Cochrane Library, EMBASE, China National Knowledge Infrastructure Database, Chinese Biomedical Literature Database, VIP Chinese Science and Technology Periodical Database, and Wanfang Data. The methodological quality of RCTs will be assessed using the Cochrane risk assessment tool. All trials included will be analyzed according to the criteria of the Cochrane Handbook. Review Manager 5.3, R-3.5.1 software will be used for publication bias analysis. Grading of recommendations assessment, development, and evaluation pro GDT web solution will be used for evidence evaluation.

**Results:**

This review will evaluate the effects of probiotics, prebiotics, and synbiotics on symptoms, lung function, asthma exacerbations, quality of life, and safety in patients with asthma.

**Conclusions:**

This review will provide clear evidence to assess the effectiveness and safety of probiotics, prebiotics, and synbiotics for asthma.

**OSF registration number:**

DOI 10.17605/OSF.IO/V7DM9.

## 1 Introduction

Asthma is a chronic condition characterized by recurrent attacks of breathlessness and wheezing, which occurs in people of all ages. Asthma is the most common chronic disease in children, with approximately 235 million people suffering from asthma as of August 2017.^[1]^ A cross-sectional study from 2012 to 2015 showed that there were 457 million adult asthma patients in China.^[2]^ The number of asthma patients may continue to grow in the next 10 years.^[1]^

In recent years, with the spread of clinical guidelines and increased access to drugs and other new treatments, asthma mortality and disease burden have been reduced.^[3–5]^ Although the currently available asthma drugs have achieved widespread success, there have been no strategies to cure asthma.^[6,7]^ Existing treatments are not always effective. There are about 10% of people who adhere to their prescribed drugs cannot control their asthma symptoms.^[8]^ Conventional treatment usually has side effects. Mood changes, transient effects, immunosuppression, growth retardation of children, and even death are associated with Inhaled corticosteroids, long-acting β agonists or Montelukast.^[9–14]^ Targeted drugs such as omalizumab are also too expensive to limit their range of applications.^[15]^ More unconventional therapies should be valued.

Probiotics are a class of active microorganisms that are beneficial to the host by colonization in the human body and altering the composition of the flora at a certain part of the host. Prebiotics are nondigestible food ingredients that have a beneficial effect on the host by selectively stimulating the growth and activity of probiotics to improve host health. Synbiotics are a combination of probiotics and prebiotics.^[16,17]^ Probiotics, prebiotics, and synbiotics can ameliorate the host immune system via gut ecosystem and may be beneficial for the treatment of allergic diseases such as asthma.^[18]^ Some animal experiments have shown that probiotics can effectively inhibit IgE production and the accumulation of eosinophils.^[19–21]^ Probiotics also show effects in the prevention and treatment of allergic diseases.^[22,23]^

Since asthma is often associated with allergies,^[1]^ we want to know if probiotics, prebiotics or synbiotics also have an effect on asthma. We decided to conduct this study after a systematic search but no similar study was founded. This systematic review has been registered on open science framework (OSF) (DOI 10.17605/OSF.IO/V7DM9).

## 2 Methods

This systematic review has been registered in OSF (https://osf.io/v7dm9/), registration number: DOI 10.17605/OSF.IO/V7DM9. Systematic review is a secondary literature research that does not require direct contact with patients, so the ethical approval and patient consent form are not necessary. We will develop and report this study in compliance with the preferred reporting items for systematic reviews and meta-analyses (PRISMA).^[24]^ The procedure of this protocol will be based on PRISMA-P guidance.^[25]^

### 2.1 Database search

Three English medical databases (Cochrane Library, PubMed, and EMBASE) and 4 Chinese medical databases (China National Knowledge Infrastructure Database, Chinese Biomedical Literature Database, VIP Chinese Science and Technology Periodical Database, and Wan Fang Data) will be systematically searched from their inceptions up to July 31, 2019. The search strategy will be based on the guidance of the Cochrane handbook.^[26]^ The search formulas of the databases are adjusted according to the following forms: (probiotic OR prebiotic OR synbiotic OR Bifidobacter^∗^ OR Lactobacill^∗^ OR Saccharomyce^∗^ OR Lactic Acid Bacteria) AND (asthma OR bronchial asthma OR allergic airway inflammation) AND (random^∗^). All relevant publications including academic dissertation and conference will be researched. There will be no language and publication date restrictions.

### 2.2 Inclusion criteria

#### 2.2.1 Types of studies

Only randomized controlled trials (RCTs) will be included.

#### 2.2.2 Types of participants

All of the participants who were diagnosed with asthma.

#### 2.2.3 Types of intervention

Probiotics, prebiotics, or synbiotics as the intervention treatment compared with blank control, placebo, or conventional treatment will be selected. Probiotics, prebiotics, or synbiotics in combination with conventional therapies compared with conventional therapies alone will also be included. All interventions will be treated for no less than 12 weeks. Basic medical treatment like oxygen therapy, infection control, and nutritional support can be used in intervention or control groups.

#### 2.2.4 Types of outcome measures

Primary outcomes: symptoms (asthma control questionnaire score and asthma control and quality of life assessment score) and lung function (forced vital capacity, forced expiratory volume in one second, and peak expiratory flow variability).

Secondary outcomes: asthma exacerbations, quality of life, and adverse event. The time endpoint of the above outcomes will be no earlier than 12 weeks after starting the medication.

### 2.3 Exclusion criteria

(1) The unrelated and duplicated documents will be deleted.
(2) Animal experiments, reviews, theoretical discussions, experience summaries, and case reports.
(3) Review articles without original data.

### 2.4 Data collection and extraction

Referring to the Cochrane collaborative network system evaluator handbook^[26]^:

(1) Importing the search results into the document management software of NoteExpress (version: 3.2, Beijing Aegean Software Company, Beijing, China);
(2) Excluding the duplicate literature using NoteExpress3.2 and excluding the unrelated articles by reading the title and abstract;
(3) Reading the full text and reserving clinical studies that meet the inclusion criteria.

Two researchers (GJ and HL) will extract the data independently using a self-developed data extraction form. The differences encountered in the process will be resolved by discussing with another team member (LWY), to determine, by agreement, the final selection of studies.

Data extraction contents will include:

(1) General information: research ID, author, title, publication status, report sources, and fund support.
(2) Methodology information: design, number of arms, random sequence generation, allocation concealment, blinding, incomplete outcome data, selective reporting, sample size calculation, and baseline comparability.
(3) Participant information: diagnostic criteria, inclusion criteria, exclusion criteria, setting, population, sample size, age, gender, and course of disease.
(4) Intervention information: name of intervention and comparation, dosage form, comparison, duration of treatment, and patient follow-up.
(5) Outcomes.
(6) Adverse events.

**Figure 1:**
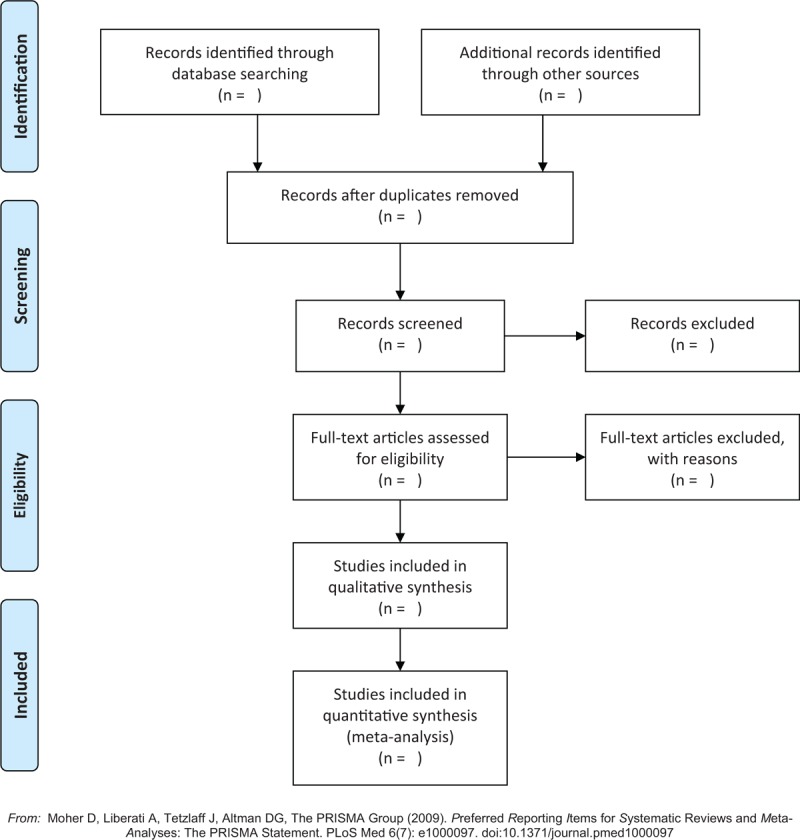
Flow chart of the selection process.

The selection process was shown in a PRISMA flow chart (http://www.prisma-statement.org/) (Fig. 1).

### 2.5 Assessment of methodological quality

Risk of bias will be assessed by the Cochrane risk assessment tool^[25]^ in 7 domains: random sequence generation, allocation concealment, blinding of the participants and personnel, blinding of outcomes assessment, incomplete outcome date, selective outcome reporting, and other bias. These domains will classify “low risk” if adequate, “high risk” if not adequate and “Unclear” if not well described by the authors in such a way that its adequacy is describable.

The 2 researchers (LH and JG) will independently assess the risk of bias for each included study. We will use “L,“ “H,“ and “U” as a code for the evaluations of the above bias risks. “L” indicating a low risk of bias, “H” indicating a high risk of bias, “U” indicating that the risk of bias is unclear. Disagreements will be resolved by discussion between all the researchers. When necessary, we will contact the study authors to inquire some missing information. Trials of high risk of bias will be considered when conducting sensitive analysis.

### 2.6 Data synthesis and analysis

Review Manager Software (RevMan, Version 5.3 for windows, The Cochrane Collaboration, Oxford, England) will be used to analyze and synthesize the outcomes. Quantitative synthesis will be done when clinical heterogeneity is not considered by at least 2 authors in discussion. Continuous variable will be described by mean difference, *P*-value and 95% confidence interval (CI). For dichotomous outcomes, we will use the relative risk, with 95% CI and *P*-values, to evaluate the efficacy and safety of probiotics/prebiotics/synbiotics. *I*^2^ test will be used to judge the heterogeneity of meta-analysis. *I*^2^ value >50% or more will be considered as an indication of substantial heterogeneity. If heterogeneity exists in the pooled studies, the data will be analyzed using a random-effects model. Otherwise, a fixed-effect model will be adopted. Sensitivity analysis or subgroup analysis will be performed if included trials are sufficient. The grouping factor for subgroup analysis will be age, asthma severity, and treatment duration. Qualitative description will be adopted if clinical heterogeneity exist.

### 2.7 Publication bias

The publication bias will be analyzed by the Egger test. The analysis software is R 3.5.1 for Windows.

### 2.8 Quality of evidence

This study evaluates the evidence according to GRADE standard, which refers grading of recommendations assessment, development, and evaluation. GRADE Pro GDT online software will be used to form the summary of findings table (SoF table).

## 3 Discussion

Probiotics, prebiotics, and synbiotics used for treating and preventing several diseases have been assessed systematically in recent years. Allergic diseases and inflammation are 2 important fields of these diseases. Intestinal flora composition plays an important role in the development of allergic diseases and airway inflammation because of its potential effects on TH1-type immunity, generation of TGF, and IgA production.^[27]^ Asthma is characterized by airway inflammation and hyper-responsiveness.^[28]^ Probiotics, prebiotics, and synbiotics were effective in suppressing both allergic and autoimmune responses, reducing allergic symptoms, and inhibiting allergic airway response in murine models of acute airway inflammation.^[29–33]^

Are probiotics, prebiotics, and synbiotics effective and safe for asthma? In order to answer this question, we searched databases but found no systematic review of RCTs published. We need more comprehensive and credible evidence to guide clinical practice. We should also assess the shortcomings of existing clinical evidence to guide future clinical trials. This study will solve the above problems.

## Author contributions

Tiegang Liu, Xiaohong Gu, and Fei Wang conceived and designed the project. Jing Guo, Ling Huang Wenyuan Li, Mei Jiang, Fei Wang, Jia Kang, Tiegang Liu, and Xiaohong Gu implemented the methods. Mei Jiang contributed analysis tools and edited review. Tiegang Liu contributed reagents/materials. Xiaohong Gu and Fei Wang revised the manuscript. All authors read and approved the final manuscript.

Ling Huang orcid: 0000-0002-0887-5544.
